# System of wheat intensification (SWI): Effects on lodging resistance, photosynthetic efficiency, soil biomes, and water productivity

**DOI:** 10.1371/journal.pone.0299785

**Published:** 2024-04-10

**Authors:** Ramesh Kumar Singh, Pravin Kumar Upadhyay, Shiva Dhar, Rajanna G. A., Vinod Kumar Singh, Rakesh Kumar, Rajiv Kumar Singh, Kapila Shekhawat, Sanjay Singh Rathore, Anchal Dass, Amit Kumar, Gaurendra Gupta, Sudhir Rajpoot, Ved Prakash, Sayantika Sarkar, Navin Kumar Sharma, Satyam Rawat, Satendra Singh

**Affiliations:** 1 ICAR-Indian Agricultural Research Institute, New Delhi, India; 2 Department of Animal Husbandry, Lucknow, India; 3 ICAR- Directorate of Groundnut Research, Regional Station, Ananthapur, India; 4 ICAR-Central Research Institute for Dryland Agriculture, Hyderabad, India; 5 ICAR-Research Complex for Eastern Region, Patna, India; 6 ICAR-Research Complex for NEH Region, Sikkim, India; 7 ICAR- Indian Grassland and Fodder Research Institute, Jhansi, India; 8 Banaras Hindu University, Varanasi, India; 9 Krishi Vigyan Kendra, Kaushambi, India; IISS: Indian Institute of Soil Science, INDIA

## Abstract

Intense cultivation with narrow row spacing in wheat, a common practice in the Indo-Gangetic plains of South Asia, renders the crop more susceptible to lodging during physiological maturity. This susceptibility, compounded by the use of traditional crop cultivars, has led to a substantial decline in overall crop productivity. In response to these challenges, a two-year field study on the system of wheat intensification (SWI) was conducted. The study involved three different cultivation methods in horizontal plots and four wheat genotypes in vertical plots, organized in a strip plot design. Our results exhibited that adoption of SWI at 20 cm × 20 cm resulted in significantly higher intercellular CO_2_ concentration (5.9–6.3%), transpiration rate (13.2–15.8%), stomatal conductance (55–59%), net photosynthetic rate (126–160%), and photosynthetically active radiation (PAR) interception (1.6–25.2%) over the existing conventional method (plant geometry 22.5 cm × continuous plant to plant spacing) of wheat cultivation. The lodging resistance capacity of both the lower and upper 3^rd^ nodes was significantly higher in the SWI compared to other cultivation methods. Among different genotypes, HD 2967 demonstrated the highest recorded value for lodging resistance capacity, followed by HD 2851, HD 3086, and HD 2894. In addition, adoption of the SWI at 20 cm × 20 cm enhanced crop grain yield by 36.9–41.6%, and biological yield by 27.5–29.8%. Significantly higher soil dehydrogenase activity (12.06 μg TPF g^-1^ soil hr^-1^), arylsulfatase activity (82.8 μg p-nitro phenol g^-1^ soil hr^-1^), alkaline phosphatase activity (3.11 n moles ethylene g^-1^ soil hr^-1^), total polysaccharides, soil microbial biomass carbon, and soil chlorophyll content were also noted under SWI over conventional method of the production. Further, increased root volumes, surface root density and higher NPK uptake were recorded under SWI at 20×20 cm in comparison to rest of the treatments. Among the tested wheat genotypes, HD-2967 and HD-3086 had demonstrated notable increases in grain and biological yields, as well as improvements in the photosynthetically active radiation (PAR) and chlorophyll content. Therefore, adoption of SWI at 20 cm ×20 cm (square planting) with cultivars HD 2967 might be the best strategy for enhancing crop productivity and resource-use efficiency under the similar wheat growing conditions of India and similar agro-ecotypes of the globe.

## 1. Introduction

The rice-wheat system (RWS) is the largest agricultural production system in the Indo-Gangetic Plains (IGP) of South Asia, spanning nearly 14 million hectares of land [1]. While being crucial for ensuring future food security and the livelihood of millions of people in South Asia [2], the RWS has encountered challenges due to declining soil health [3] depleting ground water resources [4], the practice of monocropping cereal-cereal systems, rising climate variability [5,6], and evolving socio-economic dynamics [7]. Hence, sustainable intensification of RWS using resource conservation technologies is urgently needed to meet the growing food demand while protecting the ecosystem services and environmental quality [6].

In India, wheat (*Triticum aestivum* L.) holds significant importance as a staple cereal food crop, cultivated across 31.5 million hectares, yielding approximately 112.2 million metric tons of grain, with an average yield of around 3562 kg per hectare [8]. Over a few decades, wheat production technologies that were developed made significant improvement in increasing crop yields. However, during this period, there has been a steep decline in crop productivity gains derived from the existing wheat technologies, which heavily rely on the intensive inputs used in production system [7]. The utilization of conventional production techniques and traditional cultivars in the high productive zone of the Western Indo-Gangetic Plains of India has resulted in a decline in crop productivity [9]. Therefore, an alternative approach must be explored in order to ensure that production of wheat in India could effectively address the present and future food and nutritional requirements of growing population while maintaining the sustainability [10]. Concurrently, some limitations posing threat to sustainable wheat production are depleted water resources, deteriorating the soil conditions, and higher cost of agricultural inputs accomplished by the conventional tillage management practices, uneven distribution of fertilizers, and inefficient irrigation methods [6,11]. Given the prevailing conditions, it is most desirable to assess an alternative approach of crop cultivation methods which can effectively address these potential challenges under the climate change scenario.

The SWI is a method that applies principles derived from the core principles of the system of rice intensification (SRI) with necessary adjustments [12]. The principles and practices are based on harnessing the productive capacities that arise from the plants possessing larger, more efficient, and longer-lasting root systems through diversity of soil microorganisms. Modification in wheat crop establishment enhances productivity and efficiency of inputs through better root system architecture (RSA) as suggested by Fradgley et al. [13]. Thus, RSA plays a crucial role in facilitating crop nutrient absorption, water acquisition, and grain yield [14]. These approaches should aim to achieve higher crop yields at reduced costs, minimize water usage, and enhance resilience to climatic stresses. The advantages that are documented in the context of system of wheat intensification have been drawn by the studies of Dhar et al. [15] and Park et al. [16]. According to Dhar et al. [15], Adhikari et al. [17] and Shah et al. [18] crops that possess larger and more efficient root biomass systems exhibit a greater resilience in the face of drought, storm damage, and other climatic hazards like lodging etc.

The consequence of lodging in wheat cultivation has been extensively acknowledged through various published documents. The loss in wheat production due to lodging is estimated as 57–80% globally [19,20]. The incidence of significant stem lodging has adverse impacts on typical canopy architecture of crops, leading to a reduced photosynthetic activity and grain yield loss [21]. Cultivation of tall cultivars, early planting practices, and the excessive application of fertilizers and irrigation water to enhance crop production are some of the key factors that contribute to wheat lodging. While system of wheat intensification (SWI) helps in enhancing the richness and action of soil microorganisms in the rhizosphere of crops, as well as within the phyllosphere of the crops, thus enabling more root growth below the ground [12,15,16,22]. However, our observations revealed a scarcity of documentation available on root studies and their impact on lodging under the SWI. Therefore, the extrapolated methodology of SWI with improved genotypes need examination to know its advantages which can be further validated in different genotypes of wheat under experimental conditions. In the light of the aforementioned information, the current study was planned to assess the morpho-physiological changes in wheat genotypes, growth attributes, crop physiological efficiency, productivity, quality and changes in soil biome under the different SWI techniques and conventional method of wheat cultivation.

## 2. Materials and methods

### 2.1. Field experimentation detail

A study was conducted at Indian Agricultural Research Institute, New Delhi [28.38° Latitude and 77.09° Longitude] in the winter (*Rabi*) season of 2014–15 and 2015–16. The soil of experimental plot was alluvial with sandy-clay-loam textural classes (sand, silt and clay 63.34, 12.25 and 24.41%, respectively) in 30 cm soil depth.

**Fig 1:**
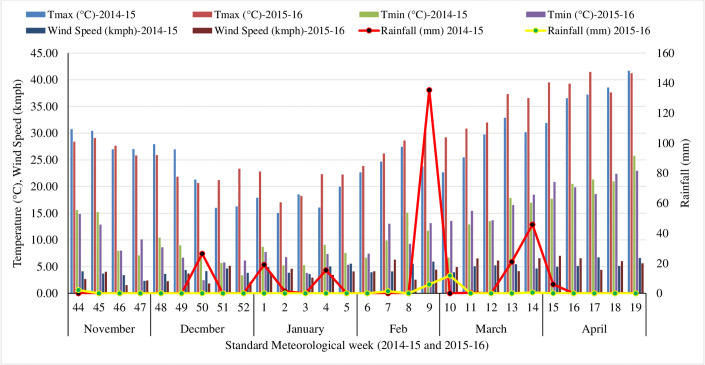
Mean weekly weather parameters during 2014–15 and 2015–16 wheat growing seasons.

At the inception of the experiment the soils had pH 7.8 (slightly alkaline), organic carbon 0.39%, available nitrogen (N) 155.4 kg ha^-1^, available phosphorus (P) 14.2 kg ha^-1^ and available potassium (K) 311.4 kg ha^-1^. The temperature during the cropping seasons was highly variable with maximum temperature ranging from 12.5–35.6°C (2014–15) and 13.9–39.5°C (2015–16), while minimum temperatures ranging from -0.90–20.2°C (2014–15) and 0.5–23.0°C (2015–16) during the cropping periods. An amount of 315.8 mm (2014–15) and 19.8 mm (2015–16) rainfall was received throughout the wheat growing season (Fig 1).

The experiment comprised of 12 treatment combinations with three cultivation methods [SWI at 20 cm × 20 cm, rectangle planting at 20 cm ×10 cm, and conventional method at 22.5 cm × continuous plant to plant spacing] in horizontal-plot, and four wheat genotypes [HD 2967, HD 3086, HD 2851 and HD 2894] fitted in the sub-plots using strip-plot-design (STP) and replicated thrice. The size of the net plot was 15.0 m^2^ (5.0 m × 3.0 m). In preparation for the field experiment, pre-irrigation was carried out, and subsequently, the field was ploughed using a tractor-drawn disc plough. After the soil achieved fine tilth conditions, harrowing was performed to further refine the soil. Conventional method of wheat cultivation was done with plant geometry 22.5 cm × continuous plant to plant spacing through seed drill. In SWI plots, single seed was placed manually in the demarcated hills of square planting at 20 cm × 20 cm and rectangular planting at 20 cm ×10 cm. The detail of agronomic management performed during the experimentation is given in the Table 1. SWI protocol developed by Dhar et al. [15] was followed in the experimentation. The check basin irrigation method was followed in wheat with 5 and 6 irrigations given during 2014–15 and 2015–16, respectively. From the net plots, wheat crop was harvested using sickles in third week of April during both the years of study.

**Table 1:** Standard agronomic management practices followed during the experimental period (2014–16).

Agronomic practices	Conventional method	SWI (20 cm × 20 cm)	Rectangle planting (20 cm ×10 cm)
Cultivars/hybrid	HD 2967, HD 3086, HD 2851 and HD 2894	HD 2967, HD 3086, HD 2851 and HD 2894	HD 2967, HD 3086, HD 2851 and HD 2894
Seed rate (kg/ha)	100 kg	25 kg	50 kg
Dates of sowing	21 November 2014 and 23 November 2015	21 November 2014 and 23 November 2015	21 November 2014 & 23 November 2015
Plot size	5.0 m x 3.0 m = 15.0 m^2^	5.0 m x 3.0 m = 15.0 m^2^	5.0 m x 3.0 m = 15.0 m^2^
Fertilizers (N, P, K kg ha^-1^)	150, 60, 60	60, 32, 40	60, 32, 40
*Trichoderma*-treatment	–	2.5 kg t^–1^ of compost	2.5 kg t^–1^ of compost
Phosphorus solubilizing bacteria application	–	6.25 kg ha^–1^	6.25 kg ha^–1^
Compost (FYM^#^)	–	2.0 t ha^–1^	2.0 t ha^–1^
Vermicompost		1.0 t ha^–1^	1.0 t ha^–1^
Weed management	Sulfosulfuron @ 20 g ha^–1^ at 25 days after sowing	Cono-weeder at 20, 30 and 40 days after sowing	Cono-weeder at 20, 30 & 40 days after sowing
Dates of harvesting	14 April 2015 and 16 April 2016	14 April 2015 and 16 April 2016	14 April 2015 and 16 April 2016

^#^FYM-Farm yard manures. The two-year average nitrogen (N), phosphorus (P), and potassium (K) content in compost was 0.55%, 0.23%, and 0.56%, respectively, while in vermicompost, it was 1.47%, 0.49%, and 1.39%.

### 2.2. Data collection and analysis

To study the leaf area index (LAI), net assimilation rate (NAR) and crop growth rate (CGR), samples were collected from a one-meter row length at four different stages i.e. 30 days after sowing [30 DAS], 60 DAS, 90 DAS and at harvest. The collected samples were initially sun-dried and consequently oven-dried at 65°C for a period of 36–48 hrs until a constant weight was achieved. For SWI (20 cm × 20 cm, and 20 cm × 10 cm) treatments, a total of 5 hills and 10 hills were sampled, respectively. The samples were oven-dried for further analysis. Leaf area meter (make: LI 3100C) was used for measurement of LAI. Then the average was worked out and it was computed as per the Eq 1.


$$Leaf\;area\;index=\frac{Leaf\;area\;({cm}^{2})}{\;Land\;area\;({cm}^{2})}$$ (1)

The CGR & NAR were worked out as per the formulae described by Watson et al. [23] and Watson [24], respectively.

The portable photosynthesis system called Infrared Gas Analyzer (IRGA) was used to measure the physiology of wheat plant during experimentation as suggested by Pandey et al. [25]. The physiological parameters, including the rate of photosynthesis, stomatal conductance, rate of transpiration and intercellular carbon dioxide concentration were determined at flowering stage of wheat using the infrared gas analyzer (IRGA).

Root samples were collected from the third row (sampling area) of the crop at the 50% flowering stage of wheat. A root auger, measuring 8 cm in diameter and 15 cm in length (with a core volume of 754.28 cm^3^) was utilized to extract root samples from the soil profile up to a depth of 0–15 cm. The core ring of the auger was carefully positioned at the base of the wheat stem in the center for each sampling. Adopting the method outlined by Costa et al. [26], the collected root samples were placed in a container equipped with sieves to separate and eliminate soil debris. To preserve the delicate nature of the roots, the samples were subsequently stored in a refrigerator at 4°C. The WIN-RHIZO system was employed for scanning and image analysis to record various root characteristics, such as root length and root volume. Following the scanning process, the root samples were dried at 60°C for 48 hours to measure the root dry weight.

### 2.3. Lodging tendency analysis

At the heading stage of wheat, the primary tillers were collected, specifically from their lower third and upper third nodes. The collected tillers underwent preparation, which involved the removal of leaf blades and sheaths, resulting in 200 mm stem samples. Subsequently, a shear test was conducted on the wheat samples using the TA.XT plus Texture Analyzer from Stable Micro Systems. The Texture Analyzer had a force capacity of 500 N with a force resolution of 0.1 g and allowed for variable test speeds in the range of 1 mm s^-1^. During the calibration and setup of the instrument, the contact and trigger forces were set at 0.2 kg each, with a target distance of 8 mm. The test generated data on force and extension at failure automatically, providing force versus displacement curves. This data was then exported using the exponent software within the system. Each wheat sample was subjected to the shear test under a loading speed of 5 mm s^-1^, and the applied force required to break the lower third and upper third nodes was measured.

### 2.4. Plant chemical analysis

Grain and straw samples were collected and subsequently dried in an oven at a temperature of 60 to 70°C until a constant weight was achieved. After drying, the samples were finely ground using a Willey Mill with stainless steel blades, producing a powder form suitable for nutrient analysis. The N content in both grain and straw was expressed as a percentage and determined using the modified Kjeldahl method [27]. To calculate the nitrogen uptake, Eqs 2 and 3 were utilized:

$${N\;uptake\;in\;grain/straw\;(kg\;ha}^{-1})=\frac{\%\;N\;in\;grain/straw\times grain/straw\;yield\;{(kg\;ha}^{-1})}{100}$$ (2)

$$Total\;{N\;uptake\;(kg\;ha}^{-1})=N\;uptake\;in\;grain\;{(kg\;ha}^{-1})+N\;uptake\;in\;straw\;{(kg\;ha}^{-1})$$ (3)

The P content in both grain and straw was determined using the vanadomolybdo-phosphoric acid yellow color method (Prasad et al., 2006). To calculate the total P uptake, the P concentration was multiplied by the dry matter yield, as indicated by Eqs 4 and 5:

$${P\;uptake\;in\;grain/straw\;(kg\;ha}^{-1})=\frac{\%\;P\;in\;grain/straw\times grain/straw\;yield\;{(kg\;ha}^{-1})}{100}$$ (4)

$$Total\;{P\;uptake\;(kg\;ha}^{-1})=P\;uptake\;in\;grain\;{(kg\;ha}^{-1})+P\;uptake\;in\;straw\;{(kg\;ha}^{-1})$$ (5)

The K content in both grain and straw was determined using flame photometer, following the method described by Prasad et al. [27]. To calculate the potassium uptake, Eqs 6 and 7 were utilized:

$${K\;uptake\;in\;grain/straw\;(kg\;ha}^{-1})=\frac{\%\;K\;in\;grain/straw\times grain/straw\;yield\;{(kg\;ha}^{-1})}{100}$$ (6)

$$Total\;{P\;uptake\;(kg\;ha}^{-1})=P\;uptake\;in\;grain\;{(kg\;ha}^{-1})+P\;uptake\;in\;straw\;{(kg\;ha}^{-1})$$ (7)

### 2.5. Soil microbial dynamics

During the maximum vegetative stage of the wheat crop, soil samples were collected from each plot using core sampler, targeting a depth of 0–15 cm in the soil profile. Field moist soil samples were used for analysis of microbiological characteristics. This evaluation included measurement of microbial biomass carbon (MBC) and soil enzyme activities, such as dehydrogenase, alkaline phosphatase (APA), and total polysaccharides. To estimate the MBC in soil samples, the method described by Nunan et al. [28] was employed. Dehydrogenase activity was estimated using the method described by Casida et al. [29]. Furthermore, the measurement of APA, representing ‘free’ enzymes was conducted following the method of Tabatabai and Bremner [30]. Soil chlorophyll was assessed using the procedure outlined by Nayak *et al*. [31]. The assay involved optical density measurements at wavelengths of 663 nm, 645 nm, and 775 nm to determine the chlorophyll ‘a’ concentration.

### 2.6. Water productivity

After irrigation, the depth of water in each plot was measured at 10 selected spots using a measuring scale. Crop water productivity (CWP) and irrigation water productivity (IWP) were computed using the expressions 8 and 9, as provided by Rajanna et al. [32].


$$Crop\;water\;productivity\;\left({(kg\;ha}^{-1}mm\right)=\frac{Grain\;yield\;({(kg\;ha}^{-1})}{Evapotranspiration\;(mm)}$$ (8)

$$Irrigation\;water\;productivity\;({kg\;m}^{-3})=\frac{Grain\;yield\;({(kg\;ha}^{-1})}{Irrigation\;water\;applied\;(m^{3})}$$ (9)

### 2.7. Statistical analysis

The recorded observations during the investigation were organized in tabular form and subjected to statistical analysis to derive valid conclusions. The data underwent analysis using the standard procedure for “Analysis of Variance” (ANOVA) as described by Gomez and Gomez [33] using IBM SPSS Statistics 24 software. The significance of treatments was assessed by ‘F’ test (Variance ratio). In all cases, the standard error of the mean was calculated. The differences in the treatment means were tested using the Least Significant Difference (LSD) at 5% level of significance.

## 3. Results

### 3.1. Growth parameter

The LAI was significantly influenced due to various cultivation methods and wheat genotypes during 2014–15 and 2015–16 (Table 2). The conventional method demonstrated the significantly higher LAI measurements at 30 (0.26), 60 (2.60), and 90 DAS (4.81) over SWI plots. While, at 120 DAS, significantly higher LAI (1.45) was observed in SWI at 20 cm × 20 cm compared to conventional method of cultivation, but it was at par with rectangle planting at 20 cm ×10 cm. Whereas cultivation methods had no significant influence on CGR and NAR at all the growth stages of wheat during both the cropping seasons (Table 2). While mean CGR at 61-90- and 91-120-days periods was influenced significantly by different cultivation methods. Adoption of SWI at 20 cm ×20 cm recorded significantly higher CGR at 61–90 days period (14.08 g m^-2^) and 91–120 days period (28.95 g m^-2^) over other cultivation methods. No variation in Leaf Area Index (LAI), as well as in Crop Growth Rate (CGR) and Net Assimilation Rate (NAR), was observed among genotypes in both study seasons (Table 2). However, the genotype HD 2967 exhibited comparatively elevated cumulative CGR and NAR during time intervals of 0–30, 31–60, and 61–90 DAS in comparison to the other genotypes.

**Table 2:** Effect of cultivation methods on leaf area index (LAI), crop growth rate (CGR) and net assimilation rate (NAR) of wheat genotypes at different growth stages (mean data of 2 years).

Treatment	LAI				CGR (g m^-2^)				NAR (g m^-2^d^-1^)			
	30 DAS	60 DAS	90 DAS	120 DAS	0–30 Days	31–60 Days	61–90 Days	91–120 Days	0–30 Days	31–60 days	61–90 Days	91–120 Days
***Cultivation methods***												
Conventional (22.5 cm × continuous plant to plant spacing)	0.26	2.60	4.81	0.90	0.74	4.34	10.93	22.89	20.26	9.53	8.43	3.15
SWI (20 cm × 20 cm)	0.20	2.26	4.74	1.45	0.72	4.91	14.08	28.95	11.57	4.92	4.38	1.30
Rectangle planting (20 cm × 10 cm)	0.21	2.34	4.77	1.42	0.73	4.81	13.32	29.40	17.91	9.10	8.22	2.60
LSD (*P*≤0.05)	0.02	0.19	0.33	0.08	NS	NS	1.32	2.85	NS	NS	NS	NS
***Genotypes***												
HD 2967	0.23	2.45	4.88	1.30	0.75	4.82	13.17	27.47	16.77	7.89	7.08	2.30
HD 3086	0.23	2.42	4.85	1.29	0.73	4.79	12.99	27.59	15.99	7.80	7.04	2.30
HD 2851	0.22	2.38	4.76	1.24	0.72	4.55	12.84	26.71	16.50	7.69	7.03	2.30
HD 2894	0.21	2.34	4.62	1.19	0.71	4.58	12.08	26.54	17.05	7.97	6.89	2.40
LSD (*P*≤0.05)	NS	NS	NS	NS	NS	NS	NS	NS	NS	NS	NS	NS

*SWI: System of wheat intensification

### 3.2. Physiological attributes

**Fig 2:**
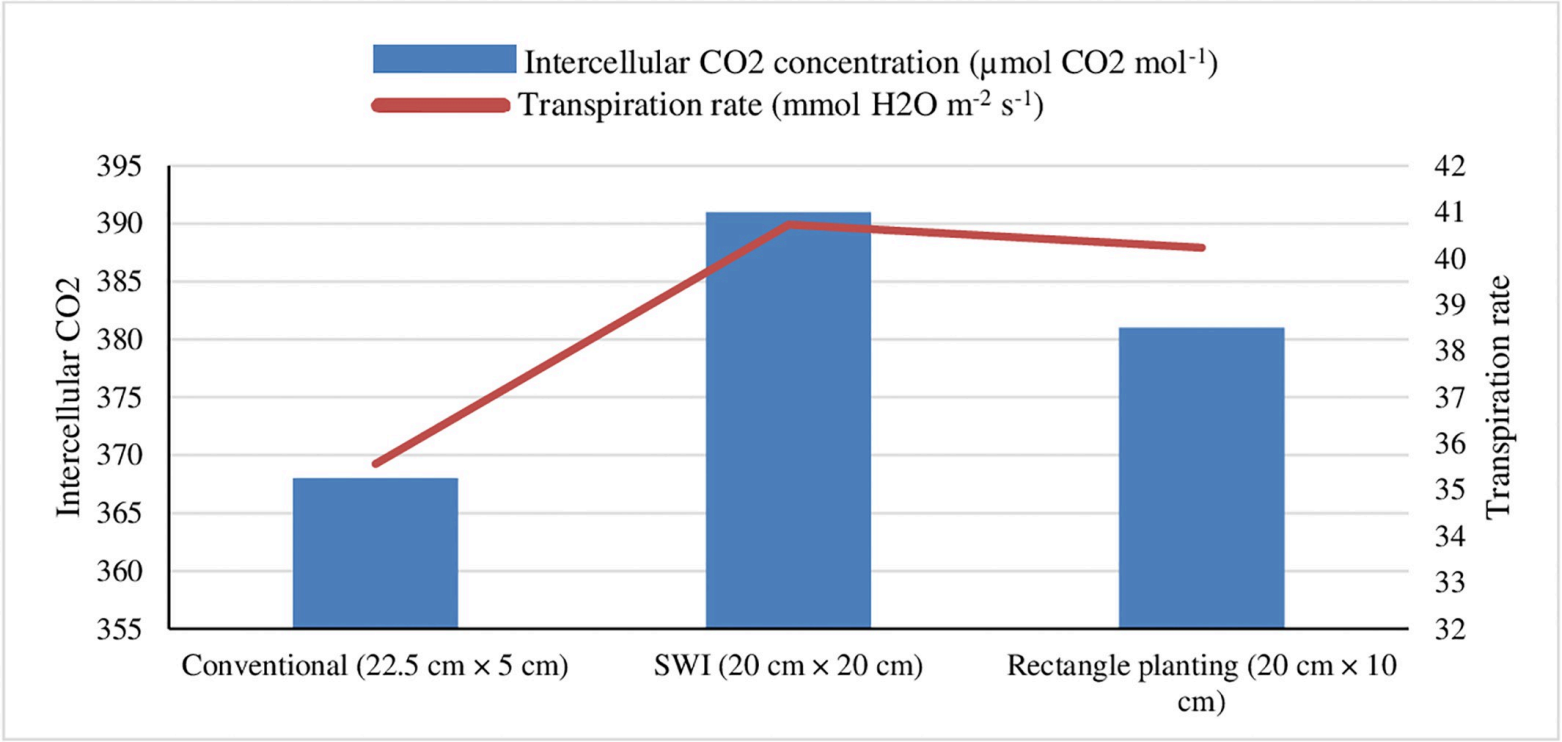
Effect of cultivation methods on intercellular CO2 concentration and transpiration rate of wheat at flowering stage (mean data of 2 years); *SWI: System of wheat intensification.

**Fig 3:**
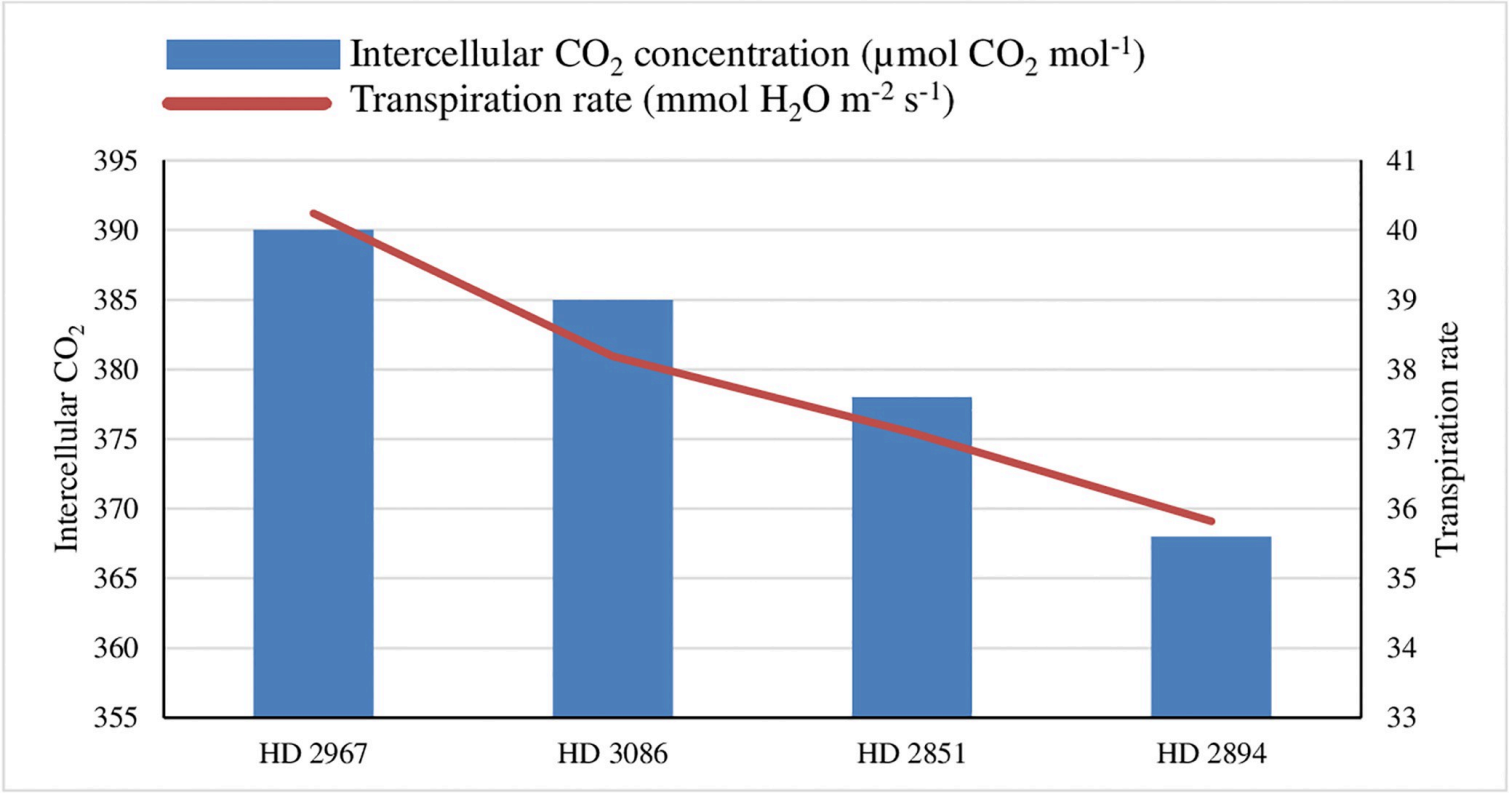
Effect of genotypes on intercellular CO2 concentration and transpiration rate of wheat at flowering stage (mean data of 2 years); *SWI: System of wheat intensification.

Adoption of the SWI techniques (Fig 2) and different wheat genotypes (Fig 3) exhibited significantly higher physiological attributes like intercellular CO_2_ concentration (Ci), transpiration (Tr) rate and stomatal conductance (Table 3) compared to the conventional method of cultivation during the experimentation. The physiological attributes like mean intercellular CO_2_ concentration (391 μmol CO_2_ mol^-1^), transpiration rate (40.73 mmol H_2_O m^-2^ s^-1^) and stomatal conductance (3.1 mol H_2_O m^-2^ s^-1^) were significantly higher with the adoption of SWI at 20 cm × 20 cm method compared to conventional method of wheat cultivation. However, no significant difference was observed between square (20 cm × 20 cm) and rectangle (20 cm ×10 cm) planting. Among the genotypes, HD 2967 exhibited elevated Ci levels (390 μmol CO_2_ mol^-1^), transpiration rate (40.24 mmol H_2_O m^-2^ s^-1^) and stomatal conductance (3.0 mol H_2_O m^-2^ s^-1^) than other wheat cultivars. However, the difference between HD 2967 and HD 3086 wheat genotypes were found to be at par with each other. Whereas wheat genotypes like HD 2851, and HD 2894 exhibited progressively lower levels of physiological attributes during study years.

**Table 3:** Effect of cultivation methods on physiological parameters of wheat genotypes at flowering stage (mean data of 2 years).

Treatment	Stomatal conductance (mol H_2_O m^-2^ s^-1^)	Net photosynthetic rate (μmol CO_2_ m^-2^ s^-1^)	PAR Transmitted [μmol (photons) m^-2^ s^-1^]	PAR Intercepted [μmol (photons) m^-2^ s^-1^]	% PAR interception
***Cultivation methods***					
Conventional (22.5 cm × continuous plant to plant spacing)	2.0	9.59	327	482	59.5
SWI (20 cm × 20 cm)	3.1	23.17	214	594	73.6
Rectangle planting (20 cm × 10 cm)	3.0	15.30	256	553	68.4
LSD (*P*≤0.05)	0.3	1.60	18.0	45.0	-
***Genotypes***					
HD 2967	3.0	17.78	353	1140	76.4
HD 3086	2.9	16.84	340	1153	77.2
HD 2851	2.5	15.51	302	1191	79.8
HD 2894	2.4	13.94	307	1186	79.5
LSD (*P*≤0.05)	0.3	0.77	15.5	39.5	-

*SWI: System of wheat intensification

A notable increase of 2.44 times in the net photosynthetic rate (NPR) was observed when adopting SWI at 20 cm × 20 cm method compared to conventional method of crop production. Similarly, the rectangle planting (20 cm ×10 cm) showed 1.52 times higher NPR than the conventional method (Table 3). Similarly, the cultivation methods have a significant impact on the transmission and interception of photosynthetically active radiation (PAR) in wheat genotypes (Table 3). The conventional cultivation method exhibited a significantly higher level of transmittance (327 μmol (photons) m^-2^ s^-1^) compared to SWI methods, while PAR interception was significantly higher under SWI plots. The PAR intercepted by wheat crop with SWI method with a spacing of 20 cm × 20 cm (594 μmol (photons) m^-2^ s^-1^) and 20 cm × 10 cm (553 μmol (photons) m^-2^ s^-1^) was 23.36% and 7.61% higher compared to conventional method, respectively. Among the genotypes, a notable increase in PAR transmittance was observed in HD 2967 (353 μmol (photons) m^-2^ s^-1^) over other genotypes. The genotypes HD 2851 (1191 μmol (photons) m^-2^ s^-1^) and HD 2894 (1186 μmol (photons) m^-2^ s^-1^) exhibited a higher percentage of PAR interception compared to other genotypes.

### 3.3. Lodging resistance

**Fig 4:**
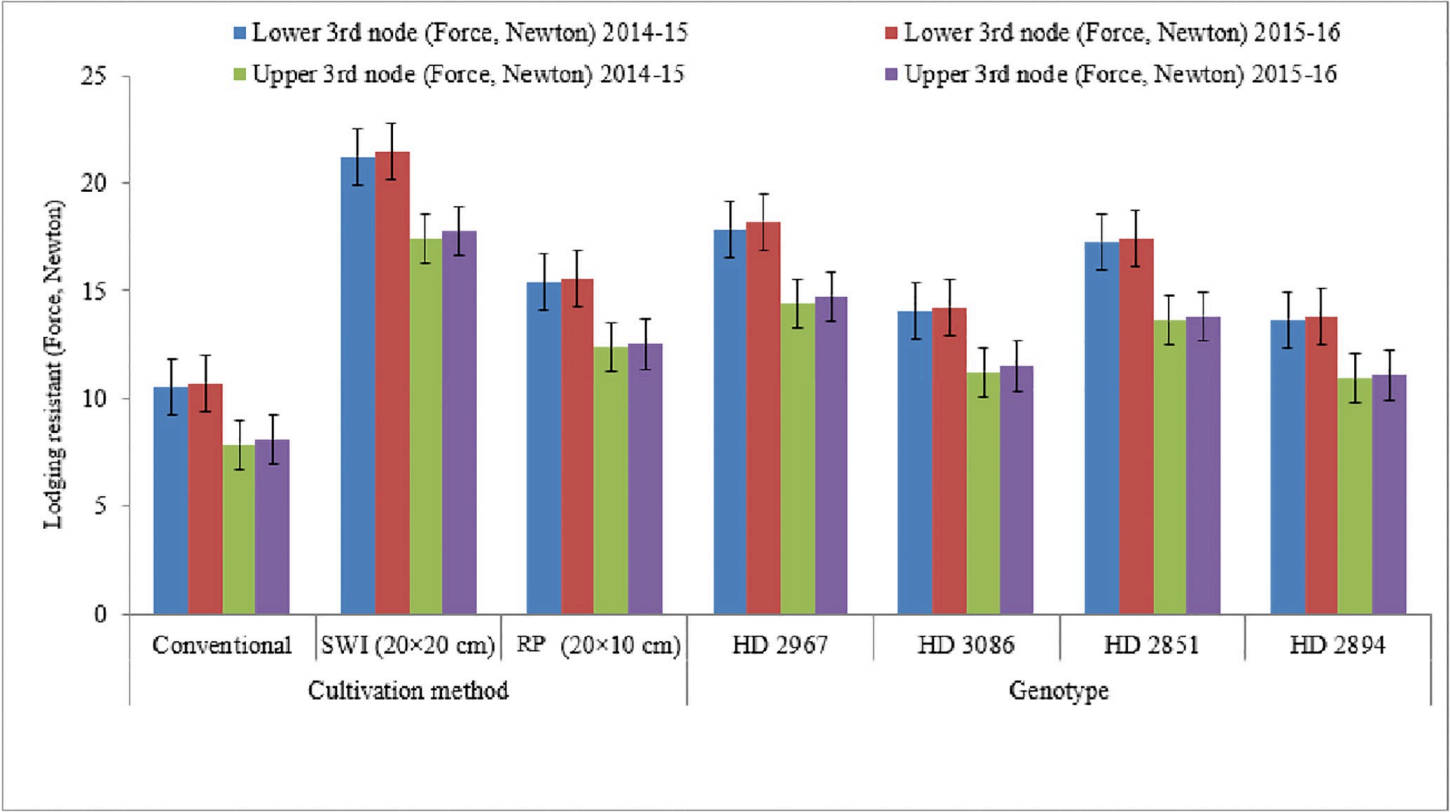
Effect of cultivation methods on lodging resistant on wheat genotypes (mean data of 2 years); *SWI: System of wheat intensification; *RP: Rectangle planting.

The lodging resistance of wheat genotypes was significantly influenced by cultivation methods in both years of the study (Fig 4). The lodging resistant capacity of the lower and upper 3^rd^ node was found to be significantly higher in SWI (20 cm × 20 cm) compared to other cultivation methods. The lodging resistance capacity showed a 2.02-fold increase under SWI (20 cm × 20 cm) for the lower 3^rd^ node and a 2.21-fold for the upper 3^rd^ node over conventional planting. Similarly, the respective lodging resistance values for SWI (20 cm × 10 cm) method were 1.37 and 1.40 folds higher than the conventional method during the years of 2014–15 and 2015–16. In terms of lodging resistance capacity, HD 2967 exhibited the highest recorded value among different genotypes followed by HD 2851, HD 3086, and HD 2894. Nevertheless, HD 2967 and HD 2851 exhibited comparable levels of resistance to upper and lower node lodging, surpassing HD 2894, and HD 3086 in this regard. The lodging resistance of wheat genotypes was found to be significantly influenced by interaction between cultivation methods and genotypes in both years (Fig 4). The wheat genotypes cultivated under both SWI and rectangle planting conditions exhibited notably greater resistance to lodging in the upper and lower 3^rd^ nodes, in comparison to those grown under conventional methods. Adoption of HD 2967 cultivar grown under SWI and rectangle planting exhibited significantly higher lodging resistance values over other genotype.

### 3.4. Crop productivity

The results of this experiment indicated that the SWI (20 cm ×20 cm) cultivation method significantly enhanced grain yield (36.9 and 41.6%), straw yield (27.8 and 30.1%), and biological yield (27.5 and 29.8%) compared to the conventional method in 2014–15 & 2015–16, respectively (Table 4). Although both SWI and rectangle planting were comparable in terms of grain yield and straw yield during 2014–15 season, the grain yield achieved under the SWI (20 cm × 20 cm) was significantly higher than that under the SWI (20 cm × 10 cm) in the 2015–16 season. Likewise, SWI approach at 20 cm × 20 cm yielded 1.96 and 3.19 t ha^-1^ grain yield, and 2.11 and 3.41 t ha^-1^ straw yield, respectively, over the conventional methods of cultivation during both the study years. Among genotypes, adoption of HD 2967 wheat genotype exhibited significantly higher grain yield (6.88 and 6.66 t ha^-1^), straw yield (11.01 and 10.65 t ha^-1^), and overall biological yield (17.89 and 17.30 t ha^-1^) compared to other genotypes of HD 2851 and HD 2894. This trend was consistent over both years, except for the first year where HD 3086 performed similarly to HD 2967. The interaction effect between cultivation methods and genotypes exhibits significant differences in terms of grain and biological yields across both years (Table 4). Combination of both SWI technique with improved genotypes of HD 2967 and HD 3086, yielded significantly compared to the other combinations tested during 2014–15 and 2015–16.

**Table 4:** Effect of cultivation methods on yield, protein content and water productivity of wheat genotypes.

Treatment	Grain yield (t ha^-1^)		Biological yield (t ha^-1^)		Protein content (%)		Total water productivity (kg ha–mm^-1^)		Irrigation water productivity (kg m^-3^)	
	2014–15	2015–16	2014–15	2015–16	2014–15	2015–16	2014–15	2015–16	2014–15	2015–16
***Cultivation methods***										
Conventional (22.5 cm × continuous plant to plant spacing)	5.31	5.07	13.59	12.98	5.31	5.07	13.59	12.98	39.06	39.09
SWI (20 cm × 20 cm)	7.27	7.18	18.75	18.50	7.27	7.18	18.75	18.50	38.82	38.84
Rectangle planting (20 cm × 10 cm)	7.11	7.00	18.35	18.01	7.11	7.00	18.35	18.01	38.80	38.87
LSD (*P*≤0.05)	0.23	0.16	0.50	0.38	0.23	0.16	0.50	0.38	NS	NS
***Genotypes***										
HD 2967	6.88	6.66	17.89	17.30	6.88	6.66	17.89	17.30	38.49	38.47
HD 3086	6.70	6.58	17.43	17.05	6.70	6.58	17.43	17.05	38.50	38.59
HD 2851	6.57	6.37	16.68	16.18	6.57	6.37	16.68	16.18	39.46	39.47
HD 2894	6.11	6.06	15.60	15.45	6.11	6.06	15.60	15.45	39.12	39.21
LSD (*P*≤0.05)	0.15	0.08	0.38	0.19	0.15	0.08	0.38	0.19	NS	NS
***Interaction of cultivation methods × genotype***										
SC1 **×**HD 2967	5.52	5.20	14.34	13.52	10.73	10.82	8.96	13.70	1.84	1.45
SC1 **×**HD3086	5.42	5.16	14.03	13.37	10.70	10.79	8.80	13.59	1.81	1.43
SC1 **×**HD2851	5.34	5.01	13.31	12.48	10.76	10.86	8.67	13.19	1.78	1.39
SC1 **×**HD2894	4.95	4.91	12.70	12.56	10.82	10.91	8.04	12.92	1.65	1.36
SC2 **×**HD 2967	7.62	7.49	19.78	19.46	10.45	10.54	14.77	28.84	3.81	3.12
SC2**×**HD3086	7.43	7.37	19.35	19.18	10.51	10.60	14.40	28.36	3.71	3.07
SC2 **×**HD2851	7.26	7.20	18.56	18.41	10.53	10.59	14.08	27.73	3.63	3.00
SC2 **×**HD2894	6.78	6.66	17.31	16.94	10.57	10.66	13.15	25.63	3.39	2.77
SC3 **×**HD 2967	7.51	7.27	19.55	18.93	10.45	10.54	14.55	27.98	3.75	3.03
SC3 **×**HD3086	7.26	7.21	18.91	18.61	10.49	10.58	14.08	27.74	3.63	3.00
SC3**×**HD2851	7.10	6.90	18.17	17.65	10.55	10.68	13.77	26.55	3.55	2.87
SC3 **×**HD2894	6.58	6.61	16.79	16.84	10.55	10.67	12.76	25.44	3.29	2.75
LSD (*P*≤0.05)	NS	NS	NS	0.67	NS	NS	NS	0.69	NS	NS

*SC1- Conventional (22.5 cm × continuous plant to plant spacing); SC2- SWI (20 cm × 20 cm); SC3- Rectangle planting (20 cm × 10 cm)

### 3.5. Crop water productivity

The cultivation methods and genotypes had a significant impact on both total water productivity (TWP) and irrigation water productivity (IWP) during 2014–15 and 2015–16 (Table 4). In the context of cultivation methods, it was observed that the use of SWI (20 cm × 20 cm) resulted in a significantly higher TWP of 39.3% when compared to the conventional method. The TWP with a SWI at 20 cm × 20 cm demonstrated comparable performance with SWI at 20 cm × 10 cm. Similarly, a notable increase in IWP was observed in SWI method (20 cm × 20 cm) compared to the conventional method, with recorded values of 3.32 kg m^-3^ and 1.59 kg m^-3^, respectively. Among the genotypes, HD 2967 genotype exhibited a notably higher TWP (17.89 kg ha-mm^-1^) and IWP of 2.83 kg m^-3^ compared to the other genotypes. However, it demonstrated comparable TWP and IWP values with HD 3086 genotype.

### 3.6. Protein yield

The cultivation methods and genotypes have significant effect on crude protein content in wheat during the present experimentation (Table 4). During the present study, it was observed that the cultivation method of SWI at 20 cm × 20 cm technique consistently resulted in higher protein yield content during 2014–15 (7.27%) and 2015–16 (7.18%) compared to other cultivation methods. It was observed that HD 2967 and HD 3086 showed significantly more protein concentration over other genotypes of HD 2851 and HD 2894.

### 3.7. Root attributes

The various wheat root attributes *viz*. length of root, volume of root, and dry mass of root were significantly inclined by SWI techniques and wheat genotypes during the present experimentation (Table 5). More root length density (RLD) was observed under SWI treatment with a spacing of 20 cm × 20 cm (4.91 cm cm^-3^), surpassing the conventional method of cultivation (3.59 cm cm^-3^). Likewise, adoption of SWI (20 cm × 20 cm) demonstrated superiority in terms of root surface area density (RSAD) (0.664 cm^2^ cm^-2^), root volume density (7.42 mm^3^ cm^-3^) and dry root mass density (2.99 mg cm^-3^) over both the conventional method of cultivation and rectangle planting (20 cm ×10 cm). However, all the root parameters under the SWI technique with spacing of 20 cm × 10 cm were comparable to that of the SWI at 20 cm × 20 cm plots. Among the genotypes, cultivating HD 2967 genotype exhibited a higher RLD (4.59 cm cm^-3^), RSAD (0.573 cm^2^ cm^-2^), root volume density (7.08 mm^3^ cm^-3^) and dry root mass density (2.97 mg cm^-3^) over other wheat genotypes. However, HD 2967 and HD 3086 exhibited statistically at par rooting parameters than HD 2851, and HD 2894 during the both the study years.

**Table 5:** Root attributes as influenced by cultivation methods and wheat genotypes (mean of 2 years).

Treatment	Root length density (cm cm^-3^)	Root surface area density (cm^2^ cm^-2^)	Root volume density (mm^3^ cm^-3^)	Dry root mass density (mg cm^-3^)
***Cultivation methods***				
Conventional (22.5 cm × continuous plant to plant spacing)	3.59	0.331	4.83	2.49
SWI (20 cm × 20 cm)	4.91	0.664	7.42	2.99
Rectangle planting (20 cm × 10 cm)	4.83	0.602	7.18	2.88
LSD (*P*≤0.05)	0.18	0.018	0.21	0.12
***Genotypes***				
HD 2967	4.59	0.573	7.08	2.97
HD 3086	4.45	0.552	6.75	2.90
HD 2851	4.40	0.505	6.08	2.66
HD 2894	4.32	0.499	5.99	2.61
LSD (*P*≤0.05)	0.12	0.016	0.23	0.10

*SWI: System of wheat intensification

### 3.8. Macronutrient uptake

The conventional method of production techniques exhibited the lower nitrogen concentrations in both grain and straw compared to SWI (Table 6). However, the concentration of N, P and K in both grain and straw remained unaffected by the different cultivation methods and genotypes. It has been observed that SWI (20 cm × 20 cm) technique leads to a greater uptake of nitrogen, P and P by both the grain (130.4, 24.7 and 32.1 kg ha^-1^), straw (54.6, 14.9 and 164.0 kg ha^-1^) and total (185.0, 39.6 and 196.1 kg ha^-1^) as compared to conventional method of wheat cultivation. However, it is worth noting that SWI (20 cm × 10 cm) production technique yields a similar level of NPK uptake with SWI (20 cm × 20 cm). Among the various genotypes studied, HD 2967 exhibited a significantly higher level of NPK uptake by grain (122.3, 23.2 and 30.2 kg ha^-1^, respectively), straw (51.3, 14.2 and 158.4 kg ha^-1^, respectively) and total uptake of 173.6, 37.4 and 188.5 kg ha^-1^, respectively compared to the other genotypes. Furthermore, HD 2967 demonstrated similar levels of NPK uptake as that of HD 3086 during the present study.

**Table 6:** Effect of cultivation methods and genotypes on NPK uptake of wheat (mean data of 2 years).

Treatment	N uptake (kg ha^-1^)			P uptake (kg ha^-1^)			K uptake (kg ha^-1^)		
	Grain	Stover	Total	Grain	Stover	Total	Grain	Stover	Total
***Cultivation methods***									
Conventional (22.5 cm × continuous plant to plant spacing)	95.8	39.7	135.5	18.0	11.0	29.0	23.4	121.0	144.4
SWI (20 cm × 20 cm)	130.4	54.6	185.0	24.7	14.9	39.6	32.1	164.0	196.1
Rectangle planting (20 cm × 10 cm)	127.4	53.3	180.6	24.1	14.8	38.9	31.5	163.5	194.9
LSD (*P*≤0.05)	4.00	1.90	5.10	0.77	0.57	1.11	0.94	4.68	5.14
***Genotypes***									
HD 2967	122.3	51.3	173.6	23.2	14.2	37.4	30.2	158.4	188.55
HD 3086	120.3	50.8	171.1	22.8	13.9	36.7	29.6	155.1	184.70
HD 2851	117.7	48.4	166.1	22.3	13.3	35.5	28.9	145.9	174.80
HD 2894	122.3	51.3	173.6	21.0	12.7	33.7	27.3	138.6	165.85
LSD (*P*≤0.05)	2.60	2.00	3.40	0.39	0.49	0.50	0.62	4.46	4.86

*SWI: System of wheat intensification

### 3.9. Soil biome

The data pertaining to the soil biological properties, specifically DHA, APA, ARA (alkaline phosphatase activity), total polysaccharides, MBC, and soil chlorophyll, subsequent to the harvest of wheat crop were highly influenced due to cultivation methods (Table 7). A notable increase in soil DHA (12.06 μg TPF g^-1^ soil hr^-1^), APA (82.8 μg p-nitro phenol g^-1^ soil hr^-1^) and ARA (3.11 n moles ethylene g^-1^ soil hr^-1^) were recorded with SWI method with a plant geometry of 20 cm × 20 cm, compared to other cultivation methods. Furthermore, these soil biological properties in the SWI method with 20 cm × 20 cm were found to be comparable over SWI method of 20 cm × 10 cm. The conventional methods yielded the lowest values of soil DHA, APA, and ARA activity. Whereas the genotype impact on DHA, APA and ARA activity were not influenced significantly during the study seasons. However, the highest levels of DHA, APA and ARA were observed in HD 2894, followed by HD 2851, HD 2967, and HD 3086. Likewise, the SWI (20 cm × 20 cm) method showed similar levels of total polysaccharides to the SWI (20 cm × 10 cm) method than conventional method of cultivation.

**Table 7:** Effect of cultivation methods and genotypes on soil biological properties in wheat (mean data of 2 years).

Treatment	DHA (μg TPF g^-1^ soil hr^-1^)	APA (μg p-nitro phenol g^-1^ soil hr^-1^)	ARA activity (n moles ethylene g^-1^ soil hr^-1^)	Total polysaccharides (mg kg^-1^ soil)	Soil microbial biomass carbon (mg kg^-1^ soil)	Soil chlorophyll (mg g^-1^ soil)
***Cultivation methods***						
Conventional (22.5 cm × continuous plant to plant spacing)	7.44	62.7	0.58	242.55	187.05	0.36
SWI (20 cm × 20 cm)	12.06	82.8	3.11	388.40	218.20	0.48
Rectangle planting (20 cm × 10 cm)	11.37	79.3	3.02	384.00	216.70	0.48
LSD (*P*≤0.05)	2.69	5.87	0.14	18.60	14.75	0.03
***Genotypes***						
HD 2967	10.27	77.5	2.26	348.55	209.40	0.45
HD 3086	10.01	76.4	2.26	339.05	208.80	0.45
HD 2851	10.30	74.2	2.21	335.20	205.75	0.43
HD 2894	10.57	71.8	2.23	330.35	206.10	0.42
LSD (*P*≤0.05)	NS	NS	NS	NS	NS	NS

*SWI: System of wheat intensification; DHA: Dehydrogenase activity; APA: Alkaline phosphatase activity; ARA: Arylsulfatase activity

The soil microbial biomass carbon (SMBC) was found to be significantly greater under SWI method, which utilised a 20 cm × 20 cm area, in comparison to the conventional method. SWI and rectangle planting methods were found to be comparable in terms of their performance in SMBC. The conventional method of production system had out yielded the lowest values of SMBC during both years of the study. Similarly, the soil chlorophyll values were statistically higher under SWI (20 cm × 20 cm) method over conventional method, with an increase of 35.2%. Nevertheless, both SWI and rectangle planting exhibited similar levels of soil chlorophyll content than conventional method of production.

## 4. Discussion

### 4.1. Weather parameter and performance of wheat crop

The weather conditions are attributed to have an impact on the performance of wheat crop (Fig 1). The data showed that during 2014–15, very high rainfall (135.4 mm) was received in January, February and March as compared to 2015–16 cropping season (19.8 mm). Heavy rainfall at crop maturity stage of wheat led to severe lodging of the crops in large areas of the North-western part of the entire country. However, the crop exhibited no lodging under the System of Wheat Intensification (SWI) production system, which involved activities such as hoeing, manure application, split application of potassium fertilizers, and wide-spaced sowing of the crops. The other weather parameters like temperature, relative humidity and sunshine hours were congenial to the wheat growth and development in the study seasons.

### 4.2. Wheat growth and productivity

In the current study, adoption of SWI with crop geometry 20 cm × 20 cm produced statistically more LAI at later stages (120 DAS) over conventional method of cultivation (Table 2). Leaf area differs with cultivation technique, and it is a limiting factor for creating exact growth in wheat. Under SWI, the demand-based nutrients applied in plant rhizosphere through organic and inorganic sources during the later stages of crop growth and development led to enhance the growth attributes in wheat. Among the genotypes, significantly higher LAI was recorded under HD 2967 genotype than others. Similarly, Gawali *et al*. [34] reported that the leaf area index of wheat didn’t show any significant variation. However, they observed that PAR interception was statistically more in both sole wheat and wheat intercrop grown under 4 m × 8 m. While in the current study, square planting in wheat under SWI production system improves the effective tillers due to better utilization of the resources like moisture, nutrients, solar radiation, and leaf orientation, which increases photosynthesis and tiller expression [35,36]. Thus, square planting also helps to reduce the weeding time and encourages mechanical weeding resulting in lower weed flora and higher growth and yield attributes in wheat crop [37,38].

The SWI production system gave higher yield (Table 4) might be due to higher yield attributes. Dhar et al. [15] reported that wheat under SWI performed superior for yield attributes, grain, straw and biological yields in comparison compared to other improved wheat cultivation methods. Moreover, studies conducted by Uphoff et al. [39], Abraham et al. [40], and Rana et al. [37] have reported a substantial increase in wheat yield, ranging from approximately 18% to 67%, at farmers’ fields through the adoption of the System of Wheat Intensification (SWI) compared to the broadcast method. Likewise, adopting SWI technique in smallholding farmers helps to get the higher yields by reducing input costs on labour, seeds, and irrigation water through enhancing elasticity to the risks of climate-change [15]. Rajanna et al. [11] also suggested that narrow row spacing of 15 cm on top of raised beds to reduce carbon and energy dynamics and increase wheat growth and production. Additionally, growth of HD 2967 genotype enhanced grain and biological yields significantly over other genotypes during the experimentation. Dhar et al. [15] opined that growing of HD 2967 genotype under direct seeded SWI (SWI-DS) produced 30% higher wheat grain yield than recommended conventional improved practice (CIP). However, in the present study, during 2014–15 due to heavy unseasonal rainfall and rise in the minimum night temperature during February-March drastically reduced the wheat yields compared to 2015–16.

### 4.3. Wheat physiology

The more interception of photosynthetic active radiation (Table 3) was noted under SWI due to more green leaf coverage per unit area. Additionally, leaf area plant^-1^ increased because of wider row arrangement than the high plant density with narrow row arrangement that exhibited under conventional method of planting. Hence, the increased foliage surface area facilitates a higher rate of transpiration under favorable temperatures ranging from 22–26°C during the wheat anthesis stage. This elevated transpiration rate contributes to greater stomatal conductance (Table 3), and thus increased the CO_2_ entry into chloroplast [41]. Likewise, Nyawade et al. [42] expressed a similar viewpoint, suggesting that broader row spacing facilitated increased solar radiation reaching the soil surface. This in turn, led to higher evaporation rates from the ground, contributing to elevated rates of transpiration in crop rows spaced farther apart. Consequently, optimal row spacing results in improved light interception and better penetration of light into the crop canopy, thereby enhancing the light utilization efficiency in crop plants [43]. The findings from the present study, using the SWI technique, align with previous documented evidence supporting this notion. Likewise, physiological attributes like Intercellular CO_2_ concentration, transpiration rate, stomatal conductance, net photosynthetic rate, and % PAR interception were recorded higher in HD 2967 over other wheat genotypes (Table 3). The stay-green wheat genotypes had lower canopy temperature, long photosynthetic duration, and higher biomass than the control, which was more significant under low N and high temperature [44].

### 4.4. Water productivity

Application of FYM + vermicompost, hoeing practice enhanced the storage capacity of water in the soil that could increase the soil moisture in SWI methods. The SWI production techniques (20 cm × 20 cm) resulted in significantly higher TWP and IWP over other cultivation methods (Table 4). These findings are associated with Dhar et al. [15] who reported that wheat sown using subsurface water irrigation in farmer’s field demonstrated a 30% reduction in water usage compared to conventional farming practices. Kumar et al. [45] recorded higher irrigation water productivity by 17.3 and 17.8% in SWI (10 cm × 10 cm) as compared to conventional line sowing. Concurrently, application of surplus supplemental irrigation (SSI) & narrow row spacing noted highest water use efficiency (WUE), while deficit supplemental irrigation (DSI) at reproductive stage with broader row spacing noted with less WUE [46]. Rajanna and Dhindwal [32] reported that the raised bed planting of wheat saved 15–35% irrigation water through higher irrigation water productivity.

### 4.5. Protein content

Protein content in wheat grain varied based on cultivation methods and genotypes in both the 2014–15 and 2015–16 seasons (Table 4). Protein content was higher under SWI (20 cm × 20 cm) and rectangle planting (20 cm × 10 cm) over conventional method during 2014–15 and 2015–16 seasons. This difference in protein content can be attributed to the combined application of organic and inorganic source of nutrients, providing the necessary nitrogen (N) at early and later stages of crop growth, which is subsequently translocated into the grains. Nitrogen, constituting up to 16% of the protein’s weight, plays a crucial role in influencing protein content. According to Jat et al. [47] the protein content in wheat showed an increase with the application of farmyard manure (FYM). Nevertheless, according to Gu et al. [48], the protein content in wheat grains showed a notable improvement with the application of potassium (K), reaching its peak at 11.1% in grains that received 124 kg K per hectare.

### 4.6. Wheat root indices

Statistically higher root surface area density, root volume density, root length density, and dry root mass density were recorded in SWI (20 cm × 20 cm) over conventional method (Table 5). Higher root growth under SWI might be due to wider spacing, application of *Trichoderma* inoculated with FYM, PSB, split application of vermicompost and K which increased the soil aeration and water holding capacity as well as decreased the soil resistance, which create amiable soil environment for root growth and development. These results were also supported by Rehman et al. [49], where they reported that RLD and RMD (root mass density) were increased significantly with the application of vermicompost in wheat. In SWI, higher root length and volume were recorded as compared to other improved wheat cultivation methods [15]. Likewise, HD 2967 was found significantly superior in respect of RLD (4.65 and 4.52 cm per cm^3^), RSAD (0.58 and 0.57 cm^2^ per cm^3^), RVD (7.18 and 6.98 mm^3^ per cm^3^) and RMD (3.01 and 2.93 mg per cm^3^) over the other genotypes during both the years, while it was on par with HD 3086. The root parameters were higher in HD 2967 might be due to their higher physiological performance and nutrient uptake which resulted in vigorous growth and balanced plant architecture leading better root growth as compared to the other genotypes.

### 4.7. Lodging resistance

Adoption of SWI (20 cm × 20 cm) exhibited 2- and 1.4-folds higher lodging resistant capacity of lower and upper 3^rd^ node over conventional method and SWI production system (20 cm × 10 cm), respectively (Fig 4). As a result of the square planting employed by SWI, the wheat is better able to resist lodging since the lower portion of the stem is well anchored. The ability of wheat plants to resist lodging in SWI may also be enhanced by a split application of K, which is well evident from the present study. Concurrently, the roots attributes are stronger when using the SWI cultivation technique and this reduces the possibility of lodging. Kirandeep et al. [50] reported that the rotavator and conventional tillage sown wheat crop lodged more than happy seeder under adverse weather conditions. Similarly, the growth of HD 2967 wheat genotype showed higher lodging resistant capacity of lower 3^rd^ (17.9 and 18.2 N) and upper 3^rd^ (14.4 and 14.7 N) over other tested genotypes (Fig 4). During the present study, lodging was observed to occur later, specifically between 25 to 30 days after anthesis. However, lodging susceptible genotypes exhibited significant reductions in grain yield as a consequence. To promote lodging-resistant cultivars, it is crucial to prioritize characteristics such as increased stem diameter and wall thickness of basal internodes. Additionally, these cultivars should have fever tillers per unit area while bearing heavy spikes [44].

### 4.8. Nutrient uptake

Cultivation methods and genotypes did not affect nitrogen, phosphorus and potassium concentration in grain in 2014–15 and 2015–16. However, adoption of both SWI techniques and HD 2894 wheat genotype recorded higher nitrogen, phosphorus and potassium content in grain and straw as compared to the other genotypes (Table 6). The uptake of nutrients is determined by the concentration of nutrients in the plant and its yield of dry matter or biomass [51]. Therefore, the higher nutrient concentration observed in wheat grain and straw may be attributed to the increased grain, straw, and biological yield, which in turn leads to a greater uptake of nutrients. The favorable soil temperature under SWI method could be the cause for faster translocation of available nutrients from soil as well as its translocation to grains [12]. Similarly, the advantage of the SWI getting N through both i.e. organic and inorganic fertilizer sources in equal amount than conventional method also noted higher NPK concentration in wheat plant [52]. Likewise, high chlorophyll content indicates that nitrogen concentration in plant tissues was maintained at a high level [53].

### 4.9. Soil biological activities

Different cultivation methods had significant effects on soil biological properties like DHA, APA, ARA activity, total polysaccharides, SMBC, and soil chlorophyll in both years of study, but wheat genotypes did not differ significantly on these soil biological properties (Table 7). Among the cultivation methods, SWI production system (20 cm × 20 cm) exhibited 1.3 to 1.6 folds higher DHA, APA, ARA activity, total polysaccharides, and SMBC and soil chlorophyll content over the conventional method. The enhanced soil biological properties observed with SWI production system could potentially be attributed to the utilization of FYM and the implementation of split applications of vermicompost. These practices have been found to augment soil moisture availability, introduce additional nutrients, and enhance organic carbon levels through the decomposition of the organic residues. The existence of a readily decomposable carbon source in soil establishes a noteworthy association between microbial activity and enzyme activity [54], as well as enhanced soil water status [55], and exhibits a positive correlation with microbial biomass carbon [56]. The practice of hoeing in SWI serves as soil mulch, effectively reducing evaporation and improving soil moisture. Consequently, this practice enhances soil enzymatic activities when compared to conventional methods. Harish et al. [57] have documented that supplementing nutrients through organic sources loaded with plant growth-promoting rhizobacteria (PGPR) leads to an augmentation in soil enzyme activities, specifically dehydrogenase activity, resulting in an improvement in soil quality in wheat.

## 5. Conclusion

As per the results obtained in the current study, it can be concluded that

- Lodging and lower input use efficiency observed in wheat cultivation have led to a significant decline in crop yields. To mitigate this problem, it is essential to implement modified agronomic practices like SWI.
- The SWI is a resource-conserving technology that has been developed to enhance the wheat crop yields by reducing the lodging effects through increased solar radiation use efficiency and resulting better crop and water productivity.
- The implementation of SWI with a planting density of 20 cm ×20 cm is having a significant impact on mitigating lodging effects by improving crop growth and physiological attributes like CGR, NAR, intercellular CO_2_ concentration (5.9–6.3%), transpiration rate (13.2–15.8%), stomatal conductance (55–59%), NAR (126–160%), and PAR interception (1.6% to 25.2%).
- Above significant improvements are noted when comparing the adoption of SWI with the conventional method of wheat establishment. Consequently, there was a substantial increase in both wheat grain yield and biological yields, with improvements of 36.9–41.6% and 27.5–29.8%, respectively.
- Simultaneously, adoption of SWI production technique in wheat resulted in an increase in DHA, APA, ARA activity, total polysaccharides, SMBC, and soil chlorophyll.
- Concurrently, growing of HD-2967 and HD-3086 wheat genotypes demonstrated notable increases in crop grain yields, as well as PAR and chlorophyll content.
- Therefore, the potential benefits of implementing SWI technique in conjunction with HD-2967 and HD-3086 genotypes need to be followed to achieve the higher yields in wheat production. This approach holds promise not only for the Indo-Gangetic plains in India but also for other similar wheat-growing regions worldwide.

## Supporting information

S1 Graphical abstract (ZIP)
