# Supplementary figures and images for: System of wheat intensification (SWI): Effects on lodging resistance, photosynthetic efficiency, soil biomes, and water productivity
Source: PLoS One. 2024 Apr 10;19(4):e0299785. doi: 10.1371/journal.pone.0299785 (PMC11006180; doi:10.1371/journal.pone.0299785)

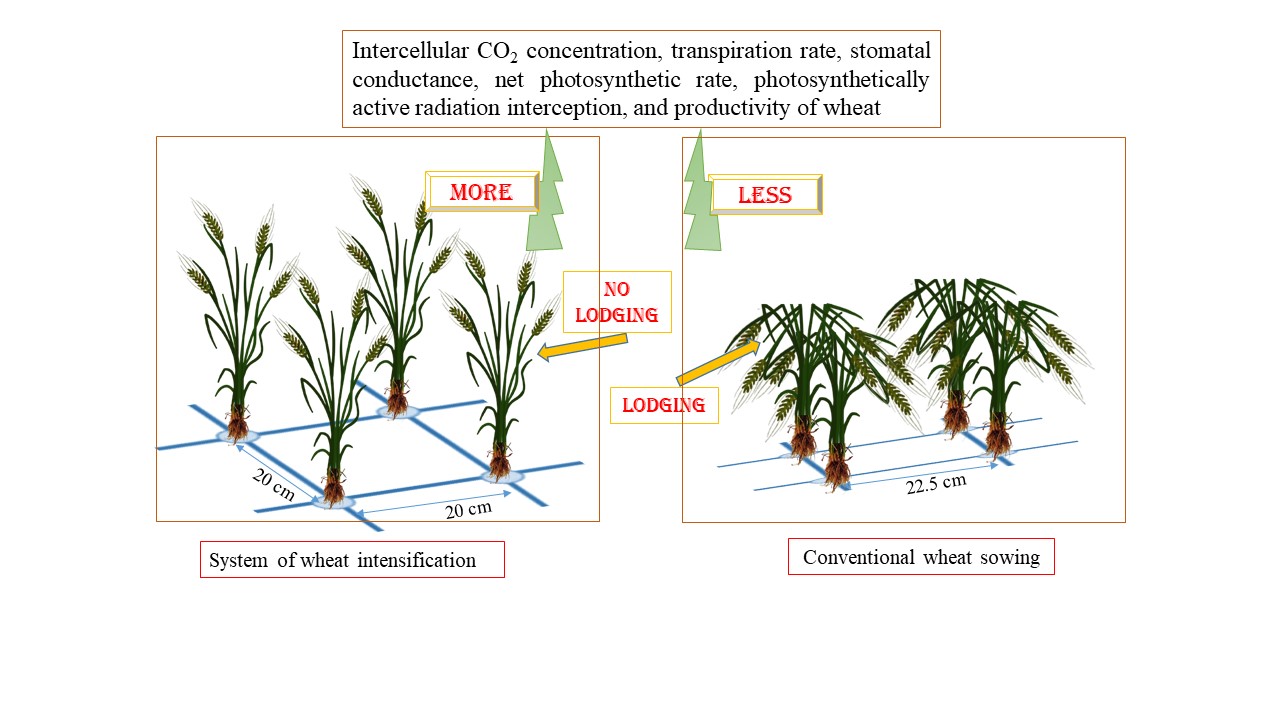

Supplement: S1 Graphical abstract — (ZIP) [file pone.0299785.s001.zip › Graphical abstract.JPG]

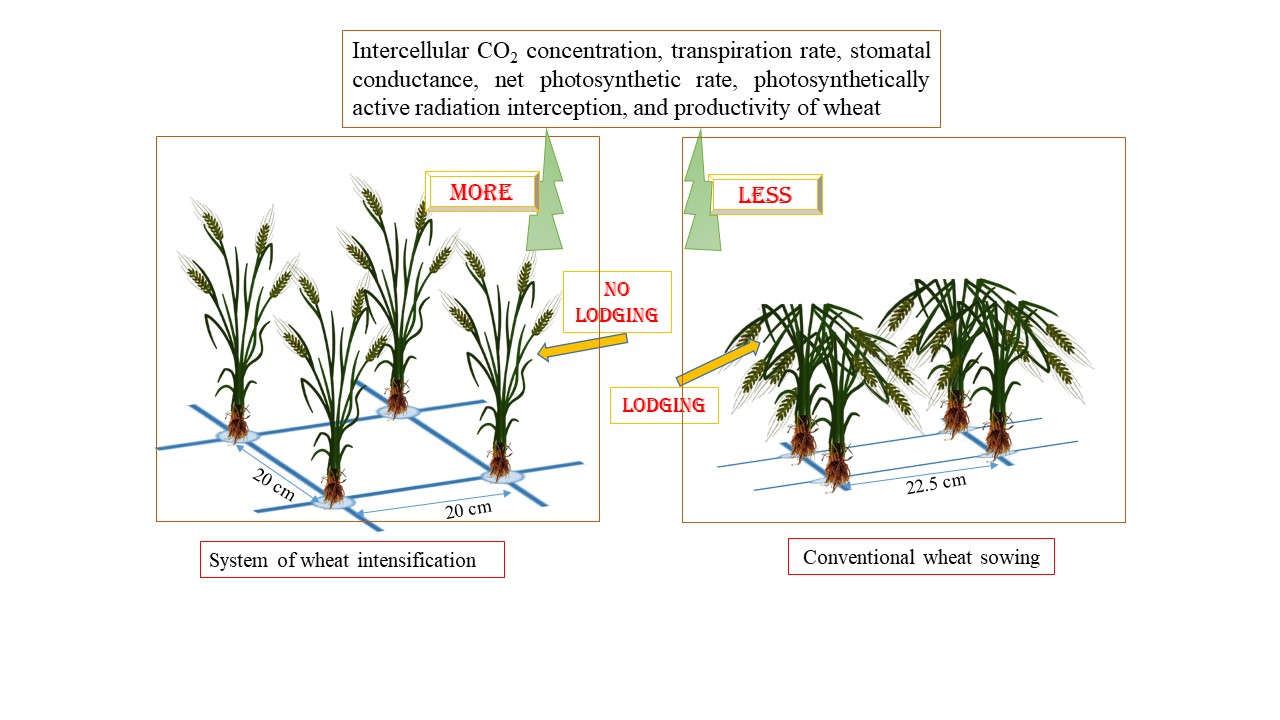

Supplement: S1 Graphical abstract — (ZIP) [file pone.0299785.s001.zip › Graphical abstract_pace.tif]
